# The relation between neck strength and psychological distress: preliminary evidence from collegiate soccer athletes

**DOI:** 10.2217/cnc-2020-0023

**Published:** 2021-05-14

**Authors:** Tara Porfido, Nicola L de Souza, Allison M Brown, Jennifer F Buckman, Brian D Fanning, James S Parrott, Carrie Esopenko

**Affiliations:** 1 School of Graduate Studies, Biomedical Sciences, Rutgers Biomedical and Health Sciences, Newark, NJ, USA; 2 Department of Rehabilitation & Movement Sciences, School of Health Professions, Rutgers Biomedical and Health Sciences, Newark, NJ, USA; 3 Department of Kinesiology & Health, Rutgers–New Brunswick, Piscataway, NJ, USA; 4 Department of Intercollegiate Athletics & Recreation, Rutgers–Newark, Newark, NJ, USA; 5 Department of Interdisciplinary Studies, School of Health Professions, Rutgers Biomedical and Health Sciences, Newark, NJ, USA

**Keywords:** cervical spine, collegiate athlete, mental health, muscle symmetry, repetitive head impacts

## Abstract

**Aim:**

To examine whether neck strength and symmetry are associated with psychological function in athletes with exposure to repetitive head impacts.

**Methods:**

Collegiate soccer (n = 29) and limited/noncontact (n = 63) athletes without a history of concussion completed the Brief Symptom Inventory 18 and assessments of isometric neck strength. Neck strength symmetry was calculated as the difference in strength between opposing muscle groups.

**Results:**

The results demonstrated that lower neck strength was associated with more symptoms of anxiety, whereas asymmetry in neck strength was associated with more symptoms of somatization and depression in soccer athletes only.

**Conclusion:**

These preliminary results suggest that greater neck strength/symmetry is related to better psychological function in athletes who have higher exposure to repetitive head impacts.

## Lay abstract

Soccer athletes experience high exposure to repetitive head impacts, which have been associated with structural and functional brain changes as well as long-term alterations in psychological function. Past work suggests that greater neck strength and symmetry help to stabilize the head when impacted, potentially limiting the negative effects of repetitive head impacts on the brain. Therefore, the authors examined the relationship between neck strength/symmetry and psychological distress in soccer athletes and limited/noncontact athletes. The authors found that lower neck strength and greater neck strength asymmetry were associated with increased symptoms of psychological distress in soccer athletes only, suggesting that lower neck strength/symmetry may be related to poorer psychological function in athletes with higher exposure to repetitive head impacts.

Approximately 150,000 National Collegiate Athletic Association athletes participate in contact sports annually in the United States [1,2] and are at a high risk of experiencing repetitive head impacts (RHIs) [3–5]. RHIs often include impacts to the head causing movement of the brain within the skull [6,7], and although RHIs often do not result in acute clinical signs or symptoms [6], cumulative exposure to RHIs can result in structural [8–12] and functional [13–15] brain changes, as well as changes in neurocognitive function [11,15–19] in active athletes. Long-term exposure to RHIs may also increase the risk of experiencing psychological problems (e.g., symptoms of depression and anxiety) in later life [20].

Soccer athletes, in particular, are at an elevated risk of exposure to RHIs [5], as their sport requires them to purposefully head the ball during both practice and games [21]. Soccer athletes are additionally exposed to unintentional head impacts (e.g., head-to-head contact during heading duels, elbow-to-head contact during tackles, ball contact to the back of the head, and head contact with the ground or goalpost) during play [21–23]. Accordingly, active soccer athletes without a symptomatic concussion show decreased white matter integrity [9], and former soccer athletes show reduced cortical thickness [24], altered brain neurochemistry [25] and cognitive impairments [24] relative to controls. Given that other contact and collision sport athletes (i.e., football and hockey athletes) show similar structural and functional brain changes as a result of RHIs [8,12,13,15] and subsequently demonstrate long-term impairments in psychological function [20], the authors sought to examine the effect of RHIs on psychological function in active soccer athletes compared to athletes participating in limited/noncontact sports.

The authors' goal was also to determine whether greater neck strength results in fewer symptoms of psychological distress in athletes with high and low expected exposure to RHIs. Laboratory studies have shown that greater neck strength can reduce the linear and rotational head accelerations that occur during purposeful heading of a soccer ball [26–28] as well as during impulsive loading (i.e., an external load producing an impulse force on the head) [29]. Studies have also shown that greater symmetry of strength between opposing muscle groups is associated with reduced linear and rotational head accelerations during soccer heading, with greater symmetry between flexor and extensor strength increasing head stability upon impact [30]. Thus, given the neck's ability to decrease head accelerations and stabilize the head during impact, greater neck strength and symmetry may be one factor that can mitigate the transfer of biomechanical forces to the brain upon impact [6,31,32].

Recent work from the authors' laboratory assessed the relation between neck strength and white matter integrity in collegiate athletes competing in soccer and limited/noncontact sports. This sample included a subset of participants from the current study who had also completed neuroimaging. The authors found that greater neck strength was associated with more intact white matter organization, but this effect was shown only in soccer athletes [33]. This suggests that greater neck strength may protect against changes to white matter tracts but is specific to athletes who are routinely exposed to RHIs as a result of participation in soccer.

In the current study, the authors sought to further understand the relation between neck strength and behavioral outcomes by examining the relationship between neck strength and psychological function in active soccer athletes with high expected exposure to RHIs and limited/noncontact sport athletes with limited to no RHI exposure. Further, as limited research shows that symmetry in strength influences head stability, exploratory analyses were also conducted to examine the relationship between neck strength symmetry and psychological function in soccer and limited/noncontact sport athletes. The authors predicted that soccer athletes would report more psychological symptoms compared with limited/noncontact sport athletes because of their higher expected exposure to RHIs. The authors also predicted that greater neck strength would be associated with fewer self-reported psychological symptoms, particularly in soccer athletes who experience higher exposure to RHIs.

## Methods

### Participants

Male and female National Collegiate Athletic Association Division III athletes participating in soccer and limited/noncontact sports (i.e., volleyball, track and field, tennis, basketball, baseball, softball and cross country) were recruited by email or in person at two campuses of a northeastern university. Data were collected from 122 participants (41 soccer and 81 limited/noncontact). Athletes who self-reported a history of concussion (n = 25) or did not complete the self-report questionnaires (n = 5) were excluded from the study, resulting in a final sample of 92 athletes (29 soccer [14 female] and 63 limited/noncontact [35 female]). Head impacts were not quantified, but participation in soccer was used as a proxy for exposure given that soccer athletes have higher expected exposure to RHIs [5,34,35]. The resultant sample included participants who also completed neuroimaging [33].

### Procedure

Participants were assessed prior to or at the start of the athletic season. Age and sport participation were collected by research personnel. Neck pain at the time of testing was determined using one question from the C3Logix graded symptom checklist (NeuroLogix Technologies, OH, USA); ‘neck pain’ was rated on a 7-point Likert scale with 0 = normal and 6 = severe [36]. Height and weight were collected by athletic personnel using a physician beam scale with height rod (Health o meter^®^ Professional Scales, model 402KL; Pelstar LLC, IL, USA) and used to determine BMI. Height and weight were not available for seven participants who had not completed preseason physical examinations at the time of testing.

**Figure 1.**
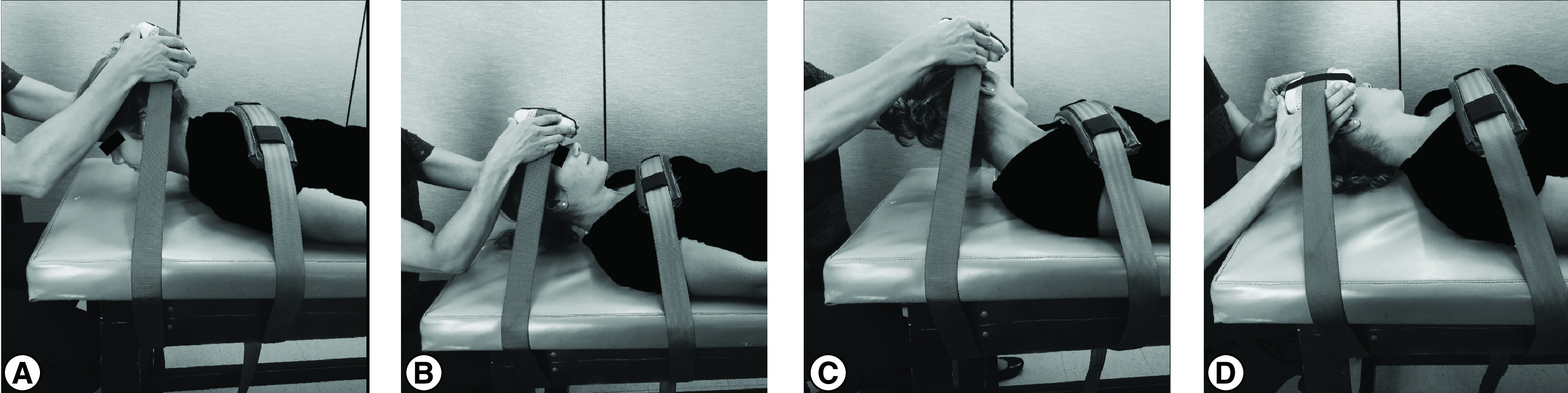
Isometric neck strength testing positions. **(A)** Extension. In the prone position, participants were instructed to perform a maximal isometric contraction in a position of mid-range extension. **(B)** Flexion. In a supine position, participants were instructed to perform a maximal isometric contraction in a position of mid-range flexion. **(C)** Right flexion in rotation. In a supine position, participants were instructed to rotate their head to the left and then asked to hold a maximal isometric contraction in mid-range flexion while maintaining left rotation. **(D)** Right rotation. In a supine position, participants were instructed to rotate their head slightly to the left and then asked to perform a maximal isometric contraction into right rotation. Left flexion in rotation and left rotation were also assessed in the same manner as that shown in **(C & D)**, but in the opposite direction.

Using the same methods described previously [33], isometric measures of neck strength were collected using a handheld dynamometer (HHD) (Lafayette Instrument Company, IL, USA) in six standardized test positions (flexion, extension, right and left rotation, and right and left flexion in rotation [37]) to test muscles important in limiting linear and rotational head accelerations (Figure 1) [26,30]. Flexion in rotation strength testing was used to isolate the sternocleidomastoid (SCM) muscle [37]. Although there are a number of muscles active during the flexion in rotation movement, this is the clinically accepted manual muscle testing position for the SCM muscle; as such, strength in this motion is referred to as SCM strength throughout the article. Strength assessments were performed with the participant lying down. To isolate movement at the neck, the torso was fixated to the plinth with a belt. In the prone position, the belt was positioned at the level of the spines of the participant's scapulae; in the supine position, the belt was positioned inferior to the participant's clavicles. Additionally, the HHD was anchored to a belt to minimize the variability associated with the manual resistance of the tester [38,39]. Three trials were performed in each test position, with a 3-s isometric hold and a 30-s rest break between each trial [28,40–42]. Participants were provided verbal cues to maintain position and constant pressure on the HHD for 3 s. The HHD recorded peak force in pounds, which was converted to kilograms and averaged across the three trials of each neck motion. All strength measurements were obtained by one of two physical therapists.

Participants also completed the Brief Symptom Inventory 18 (BSI-18) [43], a self-report questionnaire to assess psychological distress. This questionnaire has been validated in collegiate athletes and those with sports-related brain trauma [44–47]. The BSI-18 includes 18 common psychological problems and asks participants to rate the level of distress that each problem has caused them over the past week on a 5-point Likert scale, with 0 = not at all and 4 = extremely (recoded to 1–5 for the present analyses). Three subscale scores (depression, anxiety, and somatization) were calculated and used in the data analyses, with higher scores indicating greater psychological distress.

### Statistical analyses

Age, BMI, neck strength, and neck strength symmetry were compared between soccer and limited/noncontact athletes using independent *t*-tests. Bonferroni-adjusted alpha levels were used to correct for multiple comparisons in the neck strength and symmetry analyses (α = 0.008 and 0.017, respectively). The responses for neck pain symptoms and BSI-18 subscales were positively skewed, and Shapiro–Wilk tests identified that normality was violated; therefore, differences in the BSI-18 subscales between soccer and limited/noncontact athletes were examined with Mann–Whitney U tests. Sex differences in neck strength were also assessed using independent *t*-tests to ensure the sample was consistent with past research [28,48–50]; however, the authors were not powered to assess the effect of sex in the multivariate models described below. All univariate analyses were performed in SPSS Statistics 25 (IBM, NY, USA).

Consistent with past research [26–28], the average peak force in kilograms for each of the six positions was used for the neck strength analyses. Muscle strength symmetry was computed as differences in average peak force between opposing muscle groups for flexor minus extensor (flexor sub extensor), left minus right rotation (left sub right rotation), and left minus right SCM (left sub right SCM). Negative values indicated that flexor strength was weaker than extensor strength, left rotation strength was weaker than right rotation strength and left SCM strength was weaker than right SCM strength. Positive values indicated the opposite weaknesses. Two limited/noncontact athletes were identified as outliers (greater than 3 standard deviations above/below the overall group means) on neck strength and were excluded from the analyses. All results are presented with the neck strength outliers removed. Since multivariate analyses could not be completed with missing data, if a BSI-18 question was missed/skipped, the average score of the answered items within that subscale was used for the missing item [51]. Average scores were used for two participants.

Partial least squares (PLS) was used to examine the association between the BSI-18 subscales and neck strength measures. PLS is a robust multivariate technique that can quantify the pattern between two sets of variables (i.e., sets comprise a series of known highly collinear variables that cannot be included separately in a classic regression) [52,53]. PLS computes the covariance between these two sets of variables (cross-block variance) and uses singular value decomposition to identify a single latent variable (LV) to express the relationship between sets of variables [52–54]. Each significant LV reported below quantifies the pattern and strength of the relationship between two sets of variables between groups [52,53]. In the current study, the LV represents a significant pattern for neck strength with BSI-18 subscales for soccer and limited/noncontact sport athletes.

The significance of each LV was assessed with permutation testing [53]. LVs were considered statistically significant if the probability of a singular value was p < 0.05 with 1500 permutations. Bootstrap estimations assessed the contribution of each of the original variables to the LV. Specifically, bootstrap sampling with 500 samples with replacement provided an estimate of standard error. The stability of each element of the LV was computed by dividing elements by their standard errors. This ratio (referred to as a bootstrap ratio) was considered significant if it was larger than 2, similar to a *z*-score (p < 0.05), which corresponds to 95% confidence limits. Correlation profiles for the associations with the LV were calculated with bootstrap-estimated 95% confidence limits. Since PLS processes all of the variables in the same analysis step, there was no need to correct for multiple comparisons. The same PLS methods were used in the authors' exploratory analyses that examined the association between the BSI-18 subscales and neck symmetry indexes in soccer and limited/noncontact athletes. All PLS statistics were computed using version 6.15 of a PLS analysis software package (www.rotman-baycrest.on.ca/pls) in MATLAB^®^ R2019a (MathWorks, MA, USA).

## Results

### Group differences in demographics, psychological function, neck strength & symmetry

**Figure 2.**
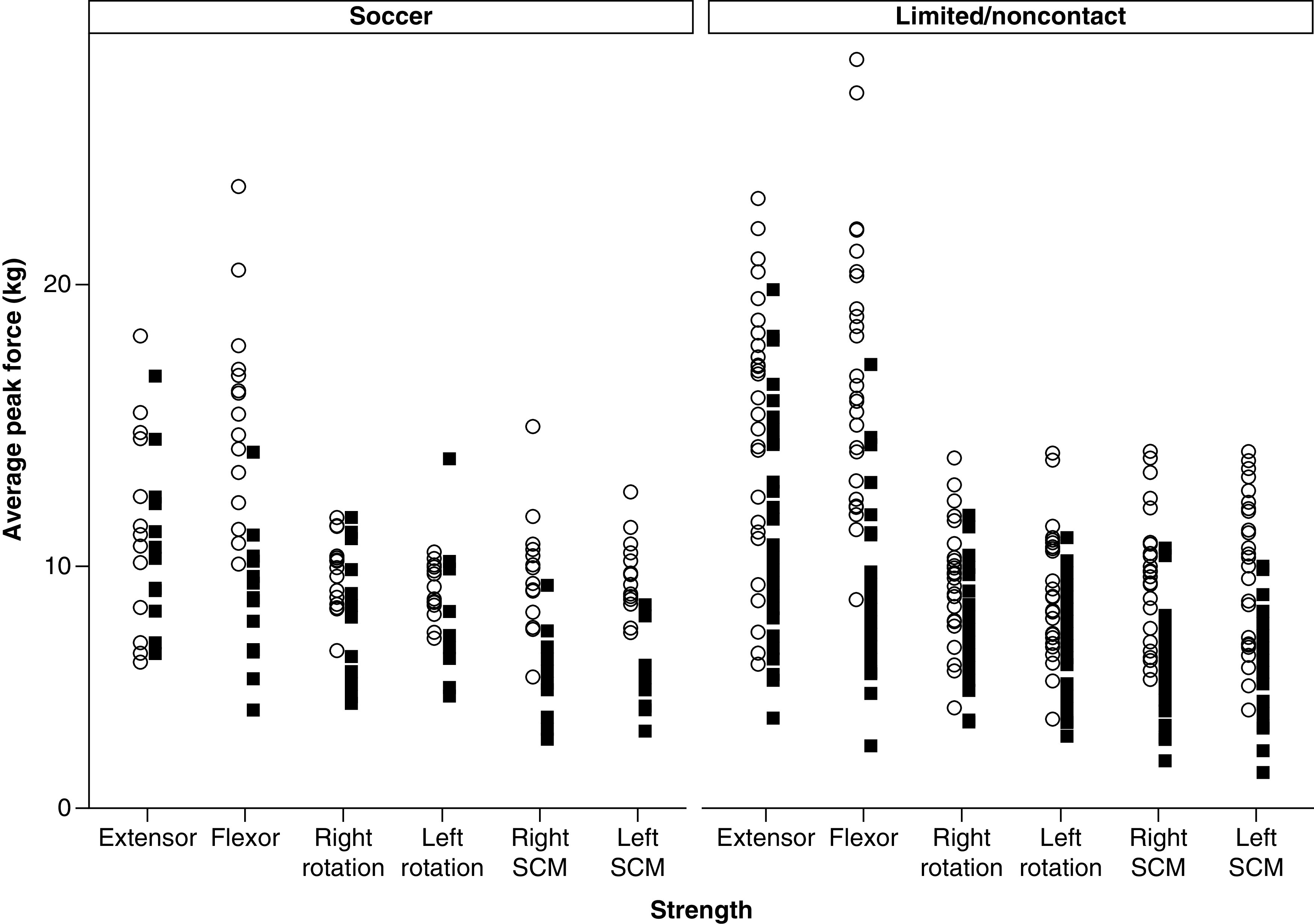
Distribution of neck strength in soccer and limited/noncontact athletes by sex. Neck strength in kilograms is plotted for male and female soccer and limited/noncontact athletes. The white circles represent male athletes, whereas the black squares represent female athletes. Distributions represent participants after the neck strength outliers greater than 3 standard deviations were removed. SCM: Sternocleidomastoid.

**Figure 3.**
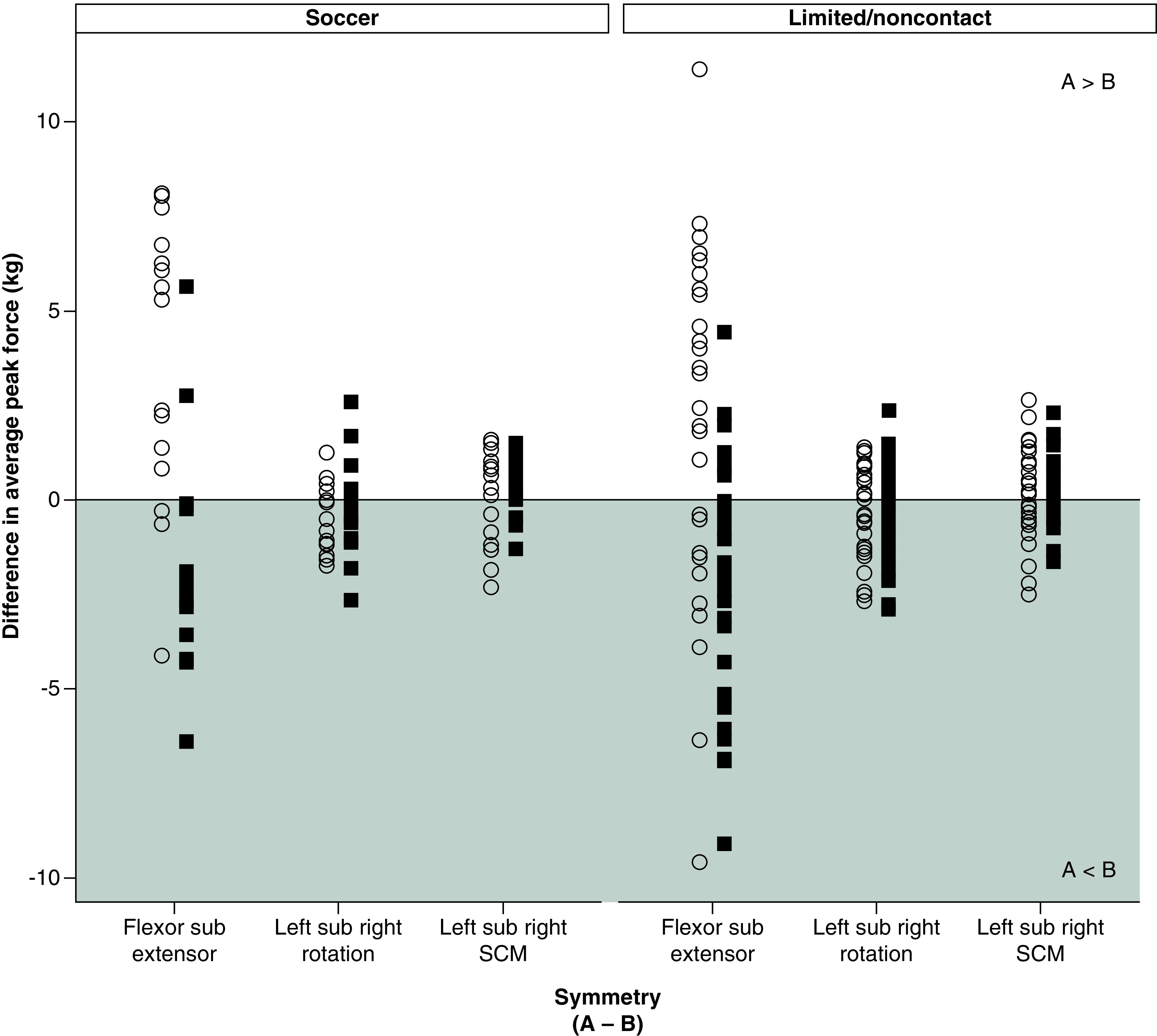
Distribution of neck strength symmetry in soccer and limited/noncontact athletes by sex. Neck strength symmetry between opposing muscle groups in kilograms is plotted for male and female soccer and limited/noncontact athletes. For each symmetry measure, the strength value listed second **(B)** is subtracted from the strength value listed first **(A).** The gray area indicates that the value of A is less than the value of B, whereas the white area indicates that the value of A is greater than the value of B. Specifically, negative values indicate that flexor strength is weaker than extensor strength, left rotation strength is weaker than right rotation strength, and left SCM strength is weaker than right SCM strength. Positive values indicate the opposite relationships. The solid black line represents zero, indicating perfect symmetry. Male athletes = white circles and female athletes = black squares. Neck symmetry outliers greater than 3 standard deviations have been removed in these distributions. SCM: Sternocleidomastoid.

There were no differences in age, BMI, neck pain, or BSI-18 subscale totals in soccer athletes compared with limited/noncontact sport athletes (p > 0.05) (Table 1). No differences were noted in neck strength or symmetry in soccer athletes compared with limited/noncontact sport athletes (p > 0.008 and > 0.017, respectively) (Table 2). Pooled across participants, male athletes demonstrated greater neck strength relative to female athletes across all test positions (p < 0.008). When strength was assessed separately in soccer and limited/noncontact sport athletes, sex differences were noted across all test positions in the limited/noncontact athletes (males > females; p < 0.008). However, similar to results seen by Bretzin *et al.*, soccer athletes did not show sex differences in strength across all test positions [28]. In the soccer athletes, males showed greater flexor, right SCM and left SCM strength (p < 0.008), but sex differences were not significant for extensor (p = 0.384), right rotation (p = 0.034), or left rotation (p = 0.076) strength. Graphical representations of the distributions of neck strength and symmetry measurements in male and female soccer and limited/noncontact athletes are provided in Figures 2 & 3, respectively. Of note, Figures 2 & 3 show distributions after outliers were removed.

**Table 1.** Demographic information and symptoms of psychological distress.

	Overall (n = 90)	Soccer (n = 29)	Limited/noncontact (n = 61)	p-value
Age (years)	19.29 ± 1.15	19.00 ± 1.23	19.43 ± 1.10	0.10
BMI (kg/m^2^)	24.62 ± 3.13	23.89 ± 2.68	24.97 ± 3.29	0.14
Neck pain^†^	0.18 ± 0.63	0.10 ± 0.31	0.21 ± 0.73	0.98
BSI-18 subscales^‡^				
Depression symptoms	6.70 ± 1.68	6.21 ± 0.49	6.93 ± 1.97	0.14
Anxiety symptoms	6.43 ± 1.16	6.34 ± 0.77	6.48 ± 1.31	0.94
Somatization symptoms	6.51 ± 1.49	6.41 ± 1.24	6.56 ± 1.60	0.40

Results are presented with neck strength outliers greater than 3 standard deviations removed. Values are mean ± standard deviation.

† Neck pain was assessed using question #3 from the C3Logix graded symptom checklist.

‡ Values obtained from BSI-18, with all responses adjusted to a 1–5 scale.

BSI-18: Brief Symptom Inventory 18.

**Table 2.** Isometric neck strength and symmetry.

	Overall (n = 90)	Soccer (n = 29)	Limited/noncontact (n = 61)	p-value
Strength^†^				
Extensor (kg)	12.37 ± 4.32	11.12 ± 3.15	12.97 ± 4.68	0.03
Flexor (kg)	12.42 ± 5.11	12.25 ± 4.42	12.50 ± 5.44	0.84
Right rotation (kg)	8.67 ± 2.08	9.01 ± 1.90	8.51 ± 2.15	0.28
Left rotation (kg)	8.25 ± 2.08	8.60 ± 1.84	8.08 ± 2.18	0.27
Right SCM (kg)	7.91 ± 2.62	7.93 ± 2.57	7.90 ± 2.67	0.96
Left SCM (kg)	8.03 ± 2.58	8.03 ± 2.21	8.03 ± 2.76	0.99
Symmetry^‡^				
Flexor sub extensor (kg)	0.05 ± 4.41	1.14 ± 4.33	-0.47 ± 4.38	0.12
Left sub right rotation (kg)	-0.42 ± 1.18	-0.41 ± 1.14	-0.42 ± 1.22	0.96
Left sub right SCM (kg)	0.12 ± 1.07	0.11 ± 1.07	0.13 ± 1.07	0.92

Results are presented with neck strength outliers greater than 3 standard deviations removed. Values are mean ± standard deviation.

† Overall strength values are significant at Bonferroni-adjusted α = 0.008.

‡ Symmetry indexes are significant at Bonferroni-adjusted α = 0.017.

SCM: Sternocleidomastoid.

### Association between neck strength & psychological function

**Figure 4.**
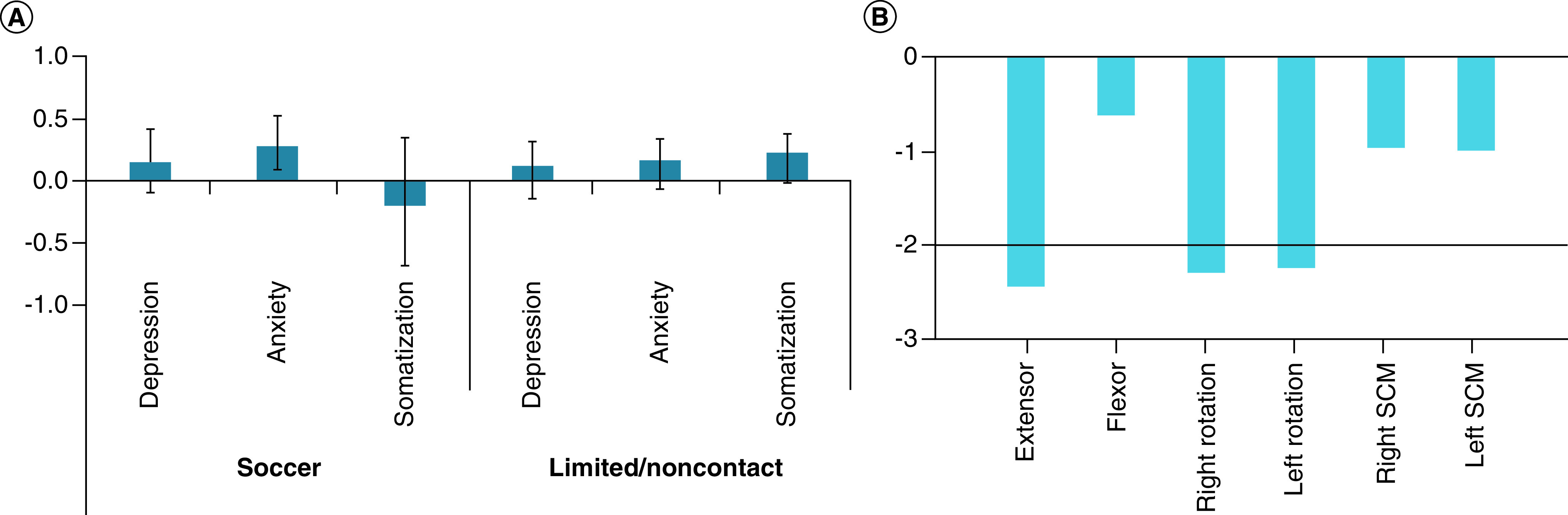
Relationship between neck strength and psychological function in soccer and limited/noncontact athletes. The results demonstrated one significant LV indicating a relationship between extensor strength, right rotation strength, and left rotation strength with anxiety symptoms. The pattern was significant in soccer athletes only. **(A)** Psychological outcomes from the BSI-18 expressed as correlations. A positive relationship is indicated when the correlation and BSR occur in the same direction. A negative relationship is indicated when the correlation and BSR occur in the opposite direction. Error bars represent 95% CIs. Error bars that cross zero were determined to be nonsignificant. **(B)** BSR for the neck strength variables. A value of -2 corresponds to a p < 0.05. The solid black line represents a significant BSR of -2. BSI-18: Brief Symptom Inventory 18; BSR: Bootstrap ratio; LV: Latent variable; SCM: Sternocleidomastoid.

One significant LV was identified indicating a relationship between neck strength and psychological function (p = 0.019), accounting for 56.1% of the cross-block variance. Specifically, lower extensor, right rotation, and left rotation strength was associated with higher scores on the BSI-18 anxiety subscale in soccer athletes only (Figure 4). No significant associations were noted for limited/noncontact sport athletes.

### Association between neck strength symmetry & psychological function

**Figure 5.**
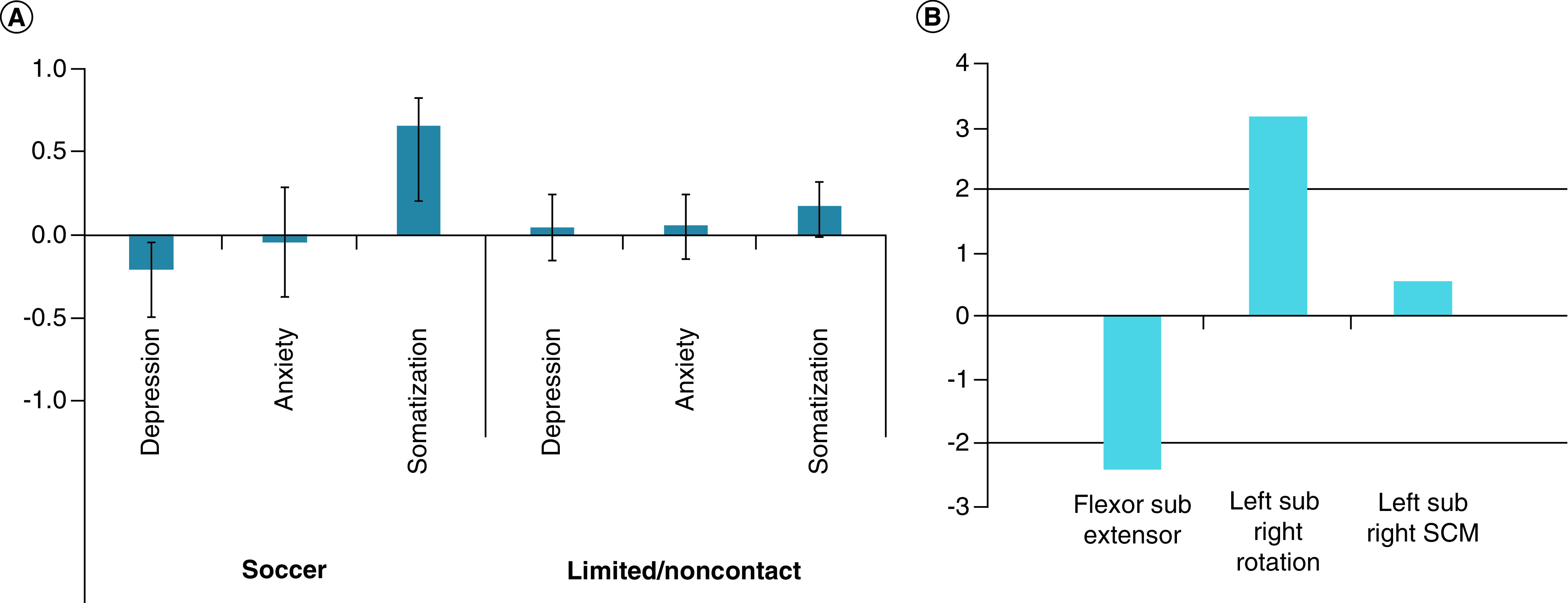
Relationship between neck strength symmetry and psychological function in soccer and limited/noncontact athletes. The results demonstrated one significant LV indicating a relationship between flexor sub extensor and left sub right rotation indexes with depression and somatization symptoms. The pattern was significant in soccer athletes only. **(A)** Psychological outcomes from the BSI-18 expressed as correlations. A positive relationship is indicated when the correlation and BSR occur in the same direction. A negative relationship is indicated when the correlation and BSR occur in the opposite direction. Error bars represent 95% CIs. Error bars that cross zero were determined to be nonsignificant. **(B)** BSR for the neck symmetry indexes. A value of ±2 corresponds to a p < 0.05. The solid black line represents a significant BSR of ±2. BSI-18: Brief Symptom Inventory 18; BSR: Bootstrap ratio; LV: Latent variable; SCM: Sternocleidomastoid.

One significant LV was identified indicating a relationship between neck strength symmetry and psychological function (p < 0.001), accounting for 78.9% of the cross-block variance (Figure 5). Specifically, when flexor strength was weaker than extensor strength and right rotation strength was weaker than left rotation strength, soccer athletes reported more somatization symptoms but fewer depression symptoms. No significant associations were noted in limited/noncontact sport athletes.

## Discussion

Head impacts typically do not result in acute observable clinical signs or symptoms [6], but cumulative exposure to RHIs is associated with impaired cognitive and psychological function in active [11,15–19] and retired [20] athletes. However, research examining the effects of RHIs on psychological function in active athletes is limited. Thus, the authors sought to examine whether active collegiate athletes with high and low risk of exposure to RHIs demonstrated different profiles of psychological distress. Although the authors had predicted that soccer athletes would report greater psychological distress given their expected higher exposure to RHIs, no univariate group differences in self-reported psychological symptoms were noted. However, these measures were collected prior to or at the start of an athletic season, when symptoms of psychological distress are expected to be low [46]. Additionally, the authors had expected that soccer athletes would have greater neck strength given the biomechanical requirements of their sport (i.e., heading the soccer ball) [55]; however, no bivariate differences in neck strength or symmetry between soccer and limited/noncontact sport athletes were found. Future research should examine whether there are changes in psychological symptoms and neck strength at the conclusion of the athletic season.

The authors also examined whether greater neck strength/symmetry was associated with fewer psychological symptoms, as the neck may have a role in reducing the long-term effects of RHIs. Recent work from the authors' group that included a reduced sample of the athletes described in the current study demonstrated that greater neck strength was associated with more intact white matter organization in soccer athletes [33]. This is consistent with past work suggesting that neck strength may limit the magnitude of force transferred to the brain upon impact by decreasing the linear and rotational head accelerations that occur when heading a soccer ball [26,28–30]. In the current study, the authors found that greater neck strength was associated with fewer anxiety symptoms in soccer athletes only, even in the absence of univariate group differences in psychological distress or neck strength/symmetry measures. The fact that the effect was only shown for symptoms of anxiety is likely related to the white matter tracts and brain regions that have the potential to be damaged by RHIs. That is, the white matter tracts within the limbic system and prefrontal cortex, regions that regulate anxiety and other emotions [56,57], as well as the white matter microstructure specifically within the amygdala are vulnerable to the shearing forces that can occur during a head impact [58,59]. Accordingly, past work has shown that RHIs are associated with altered white matter integrity in the cingulum, corpus callosum and amygdala [9,12,60,61], suggesting that structures involved in emotional regulation may be affected by RHIs. Additionally, in the authors' recent work showing an association between neck strength and white matter organization in soccer athletes, white matter tracts specifically associated with neck strength included tracts that are important in regulating anxiety, such as the uncinate fasciculus [33,62]. Together, these results provide preliminary evidence that neck strength is related to both white matter organization and psychological function in soccer athletes. Thus, these results suggest that greater neck strength may help to stabilize the head during impact and reduce the linear and rotational head accelerations that occur during RHIs. This in turn reduces the potential for neural damage, particularly in regions involved in emotion regulation, resulting in fewer symptoms of psychological distress. However, these exploratory results are limited to a small sample of soccer athletes and need to be replicated in a larger group of athletes.

Additionally, the exploratory symmetry results indicate associations between neck strength symmetry and psychological function in soccer athletes only. Specifically, when extensor strength was weaker than flexor strength, more depression symptoms were reported, but when flexor strength was weaker than extensor strength, more somatization symptoms were reported. Of note, the ratios of flexor to extensor strength seen in the authors' population differ from what would be expected based on the previous literature [30,48,63,64]. This may be explained by the positioning the authors used during the neck strength assessment protocol, whereby the participants were positioned at mid-range positions, whereas other work has assessed athletes in a more neutral posture [30,48]. Despite these differences in testing protocols and strength outcomes, the association between asymmetry and psychological function parallels evidence that asymmetry in flexor to extensor strength is associated with greater linear and rotational head accelerations upon impact [30]. Since the neck flexors and extensors are important in concentrically and eccentrically controlling the head during impacts (e.g., headers or collisions) [26,65], this would suggest that when there is an asymmetry in strength, the head is less stable, resulting in greater movement of the brain within the skull upon impact. Similarly, when left rotation strength was weaker than right rotation strength, more depression symptoms were reported, but when right rotation strength was weaker than left rotation strength, more somatization symptoms were reported in soccer athletes only. Although past research has not examined rotational strength asymmetries, rotational head accelerations can produce shearing forces that can result in diffuse axonal injury [66,67]. Thus, asymmetries in strength may make the head less stable when impacted, resulting in more movement of the brain within the skull. This likely results in changes in brain structure and function that can lead to more psychological symptoms. Importantly, although asymmetry in flexor/extensor strength and left/right rotation strength impacted BSI-18 subscales differently, it is possible that greater overall symmetry across muscle groups may result in a reduction in depression and somatization symptoms in athletes with high exposure to RHIs. However, further research is warranted to address why depression and somatization symptoms were specifically related to neck strength asymmetry.

### Limitations

Although these preliminary data are promising, there are some limitations of this work. All measures were collected at the preseason assessment, when psychological symptoms are expected to be low [46], which is consistent with what the authors saw in their sample. Thus, this research should be replicated at a time point when athletes are expected to have a higher symptom burden, such as post-concussion or directly following the conclusion of an athletic season. Additionally, the authors did not quantify individual head impact exposure; instead, athletes were grouped by sport participation as a proxy to identify athletes with high expected exposure to RHIs (soccer athletes) relative to athletes with low expected exposure to RHIs (limited/noncontact athletes). However, sport participation is a gross measure of RHI exposure and may not adequately characterize individual athletes' head impact exposures. Additional variables such as player position can also influence exposure [68–70], and thus future research should consider including other individualized exposure measures to further assess the relationship between neck strength and psychological outcomes associated with RHIs. Furthermore, sex differences in neck strength [28,71] and baseline concussion symptom scores [72,73] are well established; however, the authors were underpowered to examine the effect of sex in these relationships. Thus, future research is needed to examine whether sex differences in neck strength have differential impacts on psychological function in male and female soccer athletes. Finally, the authors' neck strength protocol includes only isometric measures of neck strength; however, research suggests that head stabilization during an impact is a complex process that may be related to anticipatory control and dynamic stabilization [29,42]. As such, future research should examine how neck strength/symmetry and muscle activation prior to impact collectively affect head stability and psychological outcomes in athletes with exposure to RHIs.

## Conclusion

Soccer athletes often experience high exposure to RHIs because of the nature of their sport. Recent work suggests that RHIs are associated with acute and long-term effects on psychological and neurocognitive function. Yet limited work has assessed psychological function in active athletes with high and low expected exposure to RHIs. The author's data show that there were no overall differences in psychological function or neck strength/symmetry between soccer and limited/noncontact sport athletes. However, it was also found that greater neck strength and symmetry may be related to better psychological function in soccer athletes. Future research should examine whether increasing neck strength and symmetry between opposing muscle groups prior to participation in contact sports is protective against alterations in psychological function.

## Future perspective

Past research has suggested that increased neck strength may be a potential mechanism for reducing the biomechanical forces associated with RHIs and increasing head stability. This, in turn, is thought to reduce the severity of acute and chronic changes in brain and behavioral outcomes. Other work from the authors' group has shown that greater neck strength is associated with better white matter organization in soccer athletes; however, no work has examined psychological function. The authors' results suggest that greater neck strength may also be associated with better psychological function in athletes with exposure to RHIs. Although this study has limitations (small sample size, no individualized exposure metrics), it does lay the groundwork to support further examination of these relationships with larger, well-characterized samples. It is the authors' hope that, collectively, these studies will help in the development of intervention research and protocols that increase the safety of sports with higher exposure to RHIs.

**Summary points**

**Background**

- Soccer athletes are at a high risk of experiencing repetitive head impacts (RHIs) because of the nature of the sport.
- RHIs in soccer athletes are associated with changes in brain structure and function and may affect psychological function.
- Greater neck strength and symmetry have been associated with reduced linear and rotational head accelerations during head impacts and therefore may limit the effect of RHIs on psychological function.

**Participants**

- Collegiate soccer athletes with high expected exposure to RHIs (n = 29) and limited/noncontact athletes with low expected exposure to RHIs (n = 63) participated in the study.
- Only participants without a history of concussion were included in the study.

**Relationship between neck strength/symmetry & psychological function**

- Lower neck strength was associated with increased symptoms of anxiety in soccer athletes only.
- No relationships were noted between neck strength and psychological function in limited/noncontact athletes.
- Exploratory results demonstrated that neck strength asymmetry was associated with symptoms of depression and somatization in soccer athletes only.

**Conclusion**

- The authors' preliminary results suggest that lower neck strength is associated with increased symptoms of psychological distress in soccer athletes.
